# Mexiletine Treatment for Neonatal LQT3 Syndrome: Case Report and Literature Review

**DOI:** 10.3389/fped.2021.674041

**Published:** 2021-08-24

**Authors:** Alena Bagkaki, Alexandros Tsoutsinos, Eleftheria Hatzidaki, Manolis Tzatzarakis, Fragiskos Parthenakis, Ioannis Germanakis

**Affiliations:** ^1^Pediatric Cardiology Unit, Department of Pediatrics, School of Medicine, University Hospital Heraklion, University of Crete, Heraklion, Greece; ^2^Department of Pediatric Cardiology and Adult Congenital Heart Disease, Onassis Cardiac Surgery Center, Athens, Greece; ^3^Department of Neonatology, School of Medicine, University Hospital Heraklion, University of Crete, Heraklion, Greece; ^4^Laboratory of Toxicology, School of Medicine, University of Crete, Heraklion, Greece; ^5^Department of Cardiology, School of Medicine, University Hospital Heraklion, University of Crete, Heraklion, Greece

**Keywords:** LQT syndrome, LQT3, mexiletine, SCN5A mutation, neonate, child, QT prolongation, case report

## Abstract

**Background:** Early diagnosis of long QT type 3 (LQT3) syndrome during the neonatal period is of paramount clinical importance. LQT3 syndrome results in increased mortality and a mutation-specific response to treatment compared to other more common types of LQT syndrome. Mexiletine, a sodium channel blocker, demonstrates a mutation-specific QTc shortening effect in LQT3 syndrome patients.

**Case Presentation:** A neonate manifested marked QTc prolongation after birth. An electrocardiogram (ECG) recording was performed due to positive family history of genetically confirmed LQT3 syndrome (SCN5A gene missense mutation Tyr1795Cys), and an association with sudden cardiac death was found in family members. The mexiletine QTc normalizing effect (QTc shortening from 537 to 443 ms), practical issues related to oral mexiletine treatment of our young patient, along with a literature review regarding identification and mexiletine treatment in infants with LQT3 syndrome are presented.

**Conclusions:** Mexiletine could be considered in the treatment of high-risk LQT3 patients already in the neonatal period in addition to b-blocker therapy. Availability of standardized commercial mexiletine pediatric formulas, serum mexiletine level analyses, and future prospective studies are needed to evaluate the potential beneficial effect of early mexiletine treatment on the incidence of future acute cardiac events in these high-risk LQT syndrome patients.

## Introduction

LQT3 syndrome incidence in neonates is estimated at 1/20,000 births, based on an LQT incidence of 1/2,000 births with LQT3 representing ~10% of total LQT cases (1). Despite of its rarity, early diagnosis of LQT3 early in life and preferably already in the neonatal period, is of paramount importance given the increased mortality and differences in treatment response associated with LQT3 syndrome. Affected LQT3 patients have a higher probability of being symptomatic in childhood [cardiac arrest or sudden cardiac death 18.6% compared to 1.7–4.2% in children and adolescents in mixed phenotype registries (2, 3)] and early infancy presenting as sudden infant death syndrome (SIDS) (4). This unfavorable disease's natural course, with events often occurring at rest or during sleep, can be modified by early introduction of LQT3 gene-specific treatment: The abnormal gain of function of mutant SCN5A sodium channels (alpha subunit of sodium channel protein NaV1.5) and the resulting prolonged myocardial cell depolarization (expressed in prolonged QTc in ECG recording) can be inhibited by sodium channel blockers such as mexiletine. This is particularly important as the effect of b-blockers (the mainstay of pharmacological treatment of the most common forms of LQT syndrome) in LQT3 patients is considered rather suboptimal (5). We present the QTc shortening effect of mexiletine treatment in a neonate with marked QTc prolongation. The neonate is the offspring of a family with genetically confirmed LQT3 syndrome associated with sudden cardiac death (SCD). Practical issues related to oral mexiletine treatment and a literature review regarding gene-specific treatment in LQT3 neonates and infants are also included.

## Case Presentation

**Figure 1:**
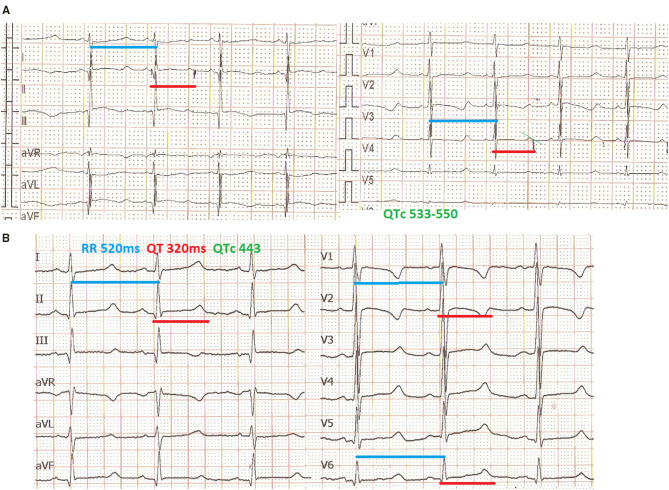
Mexiletine QTc shortening effect. ECG in the first day of life with QTc >530 ms (recording speed 25 mm/s) **(A)**, ECG at age of 3 months, under mexiletine and propranolol treatment with QTc <450 ms (recording speed 50 mm/s) **(B)**.

**Figure 2:**
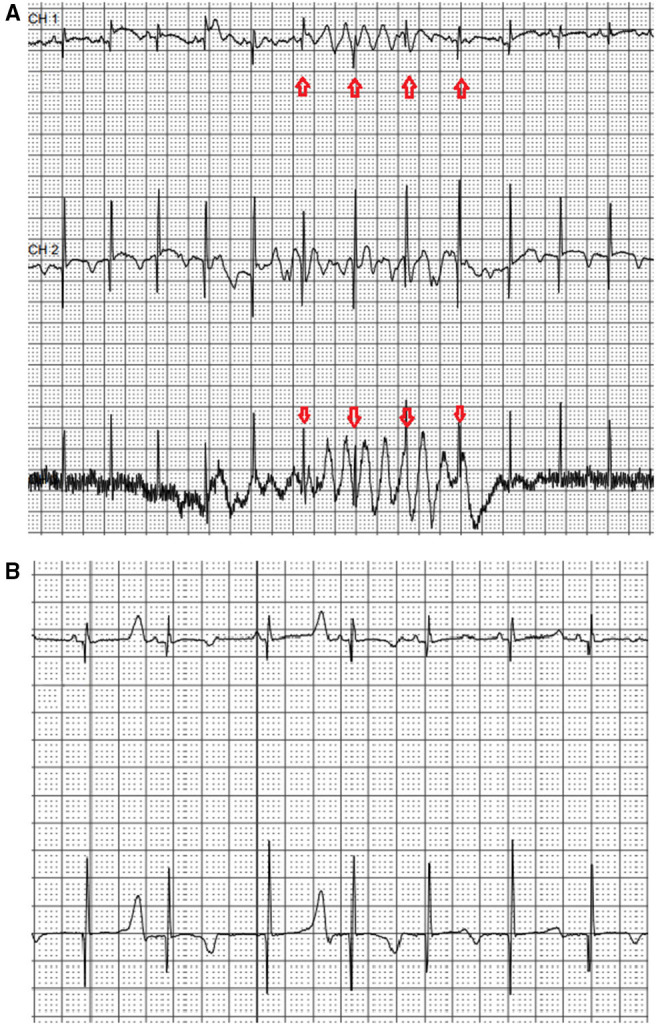
Neonatal 24-h ECG recording. Recording artifacts simulating ventricular arrhythmia -arrows **(A)** and presence of T wave alternans **(B)** during the first day of life in 24-h ECG recording.

An electrocardiogram performed in the first day of life in an asymptomatic term female neonate (38+ weeks, spontaneous birth, BW 3.4 kg) due to family history of LQT3 syndrome revealed marked QTc prolongation with late appearing T waves, persisting in repeated ECG after 24 h (QTc values 530-550, Figure 1A). Regular fetal echocardiograms during fetal life ruled out the presence of arrhythmias, while in repeated postnatal 24-h ECG recordings, only artifacts resembling ventricular tachycardia, as well as T wave alternans were documented (Figures 2A,B, respectively).

**Figure 3:**
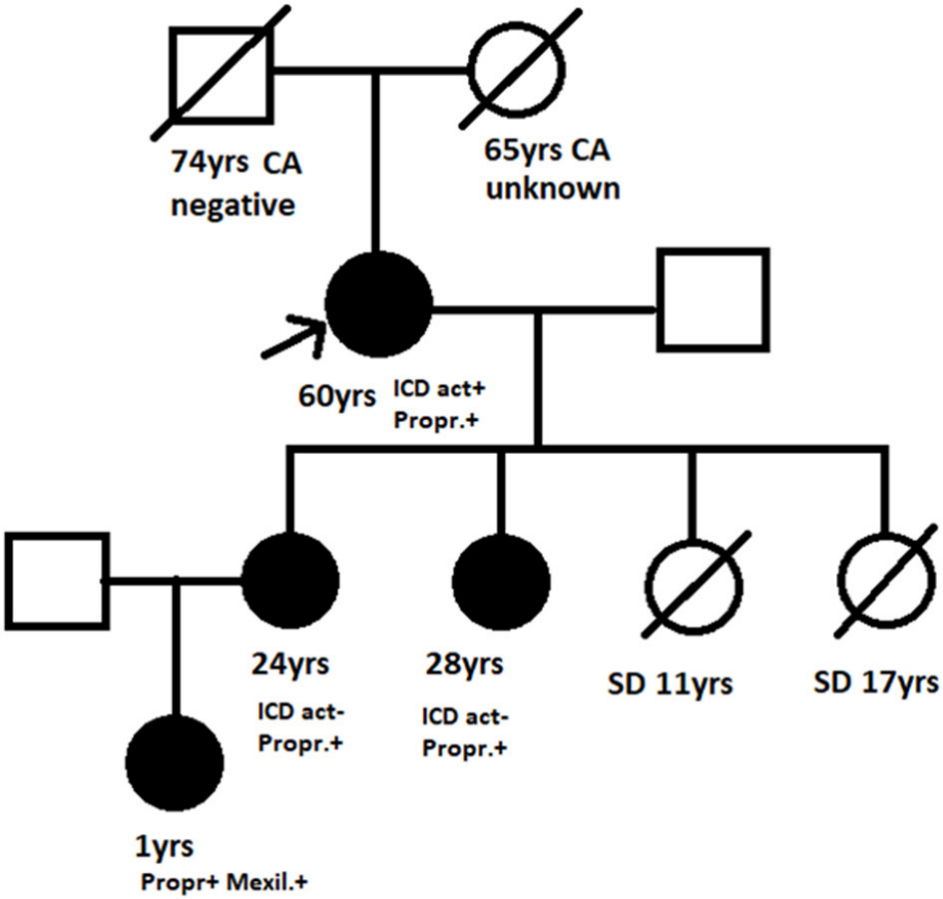
Family pedigree. Arrow, Proband; black filled symbols, Affected Y1795C mutation carrier; SD, Sudden death; CA, Cancer; negative/unknown, Y1795 mutation status; ICD, Intracardiac defibrillator; act ±, History of appropriate activation yes/no; Propr.+, Mexil.+, Propranolol/mexiletine treatment.

The presence of an SCN5A missense mutation on the C-terminus of the SCN5A gene (Tyr1795Cys) was first detected in the maternal grandmother (proband) and her two daughters (including the neonate's mother) following detailed family cardiac evaluation for sudden cardiac death (two maternal sisters died at rest at the age of 11 and 17 years, respectively) (6). Affected family members have the LQT3 ECG phenotype and are currently on propranolol and primary intracardiac defibrillator (ICD) prophylaxis (Figure 3).

The neonate was placed on combined propranolol and mexiletine oral treatment starting from the second and third day of life, respectively, with gradual dosage increase (propranolol from 1 to 2 mg/kg/d within 1 week, mexiletine from 1.8 to 4.3 mg/kg/d within the first week followed by 5 mg/kg/d in the following 2 weeks). Mexiletine administration was associated with QTc shortening on 12 lead ECGs already at a daily dosage of 4 mg/kg/d with normalization of QTc values (<460 ms) observed at a dosage of 6 mg/kg/d (Figure 1B). The QTc values manually measured from 24-h ECG recordings showed marked variability, with the highest values observed at the lowest heart rate and vice versa (Table 1).

**Table 1:** Mexiletine dosage, serum levels, and QTc values.

**Patient's age**	**Mexiletine dosage^a^ (mg/d)**	**Mexiletine dosage^a^ (mg/kg)**	**Mexiletine serum levels^b^ (ng/ml)**	**Mexiletine custom suspensionn concentration^c^**	**ECG QTc auto^d^**	**ECG QTc manually^e^**	**24-h ECG minimum QTc^f^**	**24-h ECG maximum QTc^f^**
1st day	None					516		
2nd day	None				522	537	422	533
2 months	24	4			462	460	432	490
3 months	36	6	455			443		
8 months	36	4	140	80%		477	433	516
12 months	54	5.4	345		425	418	440	504
15 months	66	6.6	545	100%	443	446	442	489

*Mexiletine dosage^a^, Daily mexiletine dosage in mg/day and mg/kg body weight; mexiletine serum levels^b^, Determined by using a liquid chromatography-mass spectrometry system; mexiletine custom suspension concentration^c^, Mexiletine concentration within the custom oral suspension as measured by liquid chromatography as percentage (%) of the reported suspension concentration; ECG QTc auto^d^, QTc auto measurement report/12-lead ECG recorder; ECG QTc manually^e^, QTc manual measurement/12-lead ECG recorder; 24-h ECG QTc^f^, QTc manual measurement/24-h ECG recorder minimum and maximum values (recorded at the highest and lowest heart rate, respectively)*.

As commercial mexiletine suspension for pediatric use is not available, a custom mexiletine aqueous suspension was prepared from mexiletine tablets (200 mg), which also had to be imported from abroad for our patient. A liquid chromatography-mass spectrometry system was used for the analytical determination of mexiletine serum levels and for confirming custom mexiletine suspension concentration (Supplementary File 1). Normalization of QTc was observed at low therapeutic levels (at the range of 500 ng/ml) while a QTc shortening effect was already present at subtherapeutic levels (Table 1).

The neonate was discharged at home following a 3-week observational period in the local and the tertiary pediatric cardiology referral center, with instructions of regular pediatric cardiology follow-up (at 3–6-month intervals based on weight gain, with 24-h ECG recordings) and avoidance of LQT prolonging drugs. The patient, currently at 15 months, remains asymptomatic, under combined propranolol (2 mg/kg/d) and mexiletine (6.5 mg/kg/d) treatment, with normal QTc values on 12-lead ECG recordings and maximum QTc values <500 ms in 24-h ECG recordings. Targeted genetic testing confirmed the presence of the familiar LQT3 mutation in our patient as well.

## Discussion

We present the normalizing effect of mexiletine on QTc values in an infant with LQT3 syndrome and marked QTc prolongation who is a carrier of an SCN5A gain-of-function missense mutation, along with the challenges of mexiletine treatment in the youngest of patients.

Soon after the SCN5A gene was first described in the early 90s (7), which encodes the a-subunit of Nav1.5 sodium channel protein and the association of LQT3 syndrome with SCN5A gain-of-function mutations, the favorable effect of the sodium channel blocker mexiletine to shorten QTc values in LQT3 was demonstrated in both animal models (8) and affected patients (9). In the latter study, were children (6–8 y). Following an oral mexiletine dose of 6–8 mg/kg, a mean QTc reduction from 535 to 445 (17% reduction) was observed within 3 h (9). Further reports confirmed the QTc shortening effect of mexiletine also in very young LQT3 infants (including a 44-day-old infant resuscitated from near-SIDS who is a carrier of an LQT3 spontaneous mutation) (4). Clinical observation of the differential response of LQT3 patients to mexiletine followed, with responders (characterized by more extended (>10%) QTc shortening and QTc values <500) manifesting a favorable clinical response on mexiletine compared to non-responders. This differential response has been attributed to mutation-specific mexiletine sodium channel effects. In this very first genotype-phenotype correlation, 4 out of 5 patients were children: 2 responders and 2 non-responders (who died at the ages of 1 and 5 years, despite being on mexiletine treatment) (10). The first LQT3 international multicenter review in pediatric LQT3 genotyped patients (<18 y) also confirmed a favorable mexiletine QTc shortening effect (at a mean dosage of 8 mg/kg/d), which was more pronounced in children with the highest QTc values >500 ms (the QTc mean value reduction was from 570 to 476 in 13 children on mexiletine) (2). Most importantly, mexiletine administration (in high doses of 11.8 mg/kg/d) effectively eliminated recurrent arrhythmias and ICD shocks in two ICD placement refractory cases. The favorable mexiletine effect was in contrast with the absence of documentation of an ICD protective effect (as primary prophylaxis) combined with a 50% ICD complication rate. A gene-specific mexiletine protective effect was similarly demonstrated, with two deaths (a 5-month-old infant on mexiletine, and a 19-year-old off treatment) among patients first presenting with fetal Torsades de Pointes (TdP).

The first study, addressing the issue whether mexiletine QTc shortening is associated with a clinical effect (reduction of arrhythmic risk) in LQT3 patients, also included 5 infants (a total of 34 patients, median age 22 y) (5). In two cases, mexiletine had already been administrated in the neonatal period (excluded from further comparative analysis). Mexiletine treatment was associated with almost 100-fold arrhythmia risk reduction. When comparing equal time intervals before and after mexiletine administration: The annual rate of arrhythmic events reduced from 10.3 to 0.7%, events per patient from 0.43 to 0.003 while the percentage of patients with arrhythmia reduced from 22 to 3% (95% CI 0–9%). All asymptomatic patients (*n* = 21) and 10/13 symptomatic patients remained asymptomatic on mexiletine treatment. Refractory arrhythmias despite mexiletine treatment were observed in 3 cases (3 deaths), all of them were diagnosed as symptomatic infants (<2 months of age) and with persistent QTc prolongation>500 ms on mexiletine. Authors concluded that the mexiletine therapeutic goal should be to obtain QTc values <500 ms for clinical prevention of sudden cardiac death (SCD) and arrhythmias in LQT3 patients (5).

In the largest international registry of LQT3 patients (406 patients with 51 SCN5A gene mutations, including the mutation observed in our patient), 15 symptomatic infants were also included. While the efficacy of b-blocker therapy could only be demonstrated for female patients (83% reduction of cardiac events), the efficacy of sodium channel blockers could not be judged given the small number of patients treated and the few events recorded. Authors concluded that in a subset of high-risk patients, including those with prior acute cardiac arrest of QTc values in the range of 500 ms, adjunctive therapy including mexiletine maybe is required (11).

The largest multicenter retrospective cohort study presenting genotype-phenotype correlations of SCN5A mutations in 442 neonates and children (including 73 infants of them 41 probands) an isolated LQT3 phenotype was present in only 47 cases (10%). The vast majority presented with other ECG phenotypes (normal ECG, isolated progressive cardiac conduction disease, overlap phenotype, isolated Brugada syndrome, in decreasing order) (12). Irrespective of ECG phenotype, patients with mutations located on the C-terminus (*n* = 110) had a lower risk (including two cases with our patient's mutation). The majority of LQT3 patients received b-blocker treatment (68%). Only 10 patients (21%) received additional sodium channel blocker treatment, of them three experienced recurrences of acute cardiac events despite combined treatment. The limited data (case reports) regarding mexiletine treatment in LQT3 neonates are presented as Supplementary File 2.

Although SCN5A mutations on the C-terminus of Nav1.5 sodium channel protein are considered a relatively benign prognosis (12), the family history of our patient (with two out of five affected family members experiencing SCD as the first manifestation) emphasizes the need of mutation-specific consultation in LQT3 patients. The family SCN5A missense mutation (Y1795C) on the C-terminal of NaV1.5 channel protein has been previously described and characterized as being associated with delayed onset of inactivation and increased expression of sustained sodium channel activity during maintained depolarization (6). According to ACMG criteria, the variant is considered pathogenic (13). It is a rare variant, having only been described in two unrelated families: The family of our patient presenting with symptomatic LQT3 syndrome (6) and a family with LQT3 associated with atrial fibrillation (14). The electrophysiological properties of the specific mutation on cellular level have been well-described (6, 15, 16).

Despite the proven effect of mexiletine to normalize QTc values in our very young patient, in accordance with the very limited available literature of mexiletine administration in neonates, mexiletine treatment in this age group is challenging: Mexiletine dosage is adjusted based on serum levels, with recommended serum concentrations in the range of 0.5–2 mcg/mL (17). Therapeutic concentrations in children may be achieved by a daily dosage of 5–15 mg/kg/day (divided into three doses) (18). However, mexiletine treatment of the youngest infants is challenging not only due to the lack of commercial mexiletine suspension but the lack of mexiletine itself in any form in several countries worldwide, as in our case. The need to import mexiletine tablets and prepare a custom suspension specific for our patient represents a major treatment drawback. Concentration of mexiletine in custom formulas might vary greatly, and verification of mexiletine concentration in custom formulas is advisable in cases of suboptimal serum levels or lack of demonstrable QTc shortening effect in LQT3 treated infants despite appropriate mexiletine dose prescription. Furthermore, as mexiletine serum levels are not routinely checked, the involvement of research laboratories performing liquid chromatography mass spectrometry for evaluating serum mexiletine level and verifying custom mexiletine suspension concentrations is needed for the safest possible mexiletine treatment of very young patients with LQT3 syndrome.

The present case report and literature review support a favorable QTc shortening effect of mexiletine in LQT3 patients when already administered from early infancy. However, the present study findings should be viewed within the present (and previous) study's limitations as well as of further issues related to effective neonatal LQT3 screening: Despite the importance of early LQT syndrome detection, universal ECG screening of all neonates has not gained wide acceptance (19). Borderline QTc prolongation observed in healthy neonates can be differentiated by documentation of spontaneous normalization of QTc values in serial ECG recordings in contrast with LQT syndrome cases presenting with persistent and often marked QTc prolongation (19). Our patient, having marked and persistent QTc prolongation underwent targeted mutation analysis for the presence of a known familiar mutation. However, for the best genotype-phenotype correlation even families and patients with known mutations should be counseled and potentially re-evaluated with further gene testing (gene panel or whole exome sequencing) for the presence of additional new described variants (or phenotype modifiers) in the light of the most updated information available. Finally, although mexiletine therapy currently represents a class IIb indication for LQT3 patients (20), prospective randomized studies are needed to demonstrate any potential survival benefit in mexiletine-treated patients, apart from the QTc shortening effect, when treatment starts early in infancy.

## Conclusions

Detection of LQT3 patients, which represents a higher risk LQT syndrome subgroup with distinct ECG features, is of paramount importance as they could benefit from early mexiletine addition to b-blocker therapy. Availability of standardized commercial mexiletine pediatric formulas and of serum mexiletine level analyses will greatly benefit the youngest LQT3 patients. Prospective randomized studies are needed to evaluate the potential beneficial effect of early mexiletine treatment on the incidence of future acute cardiac events in these high-risk LQT syndrome patients.

## Data Availability Statement

The raw data supporting the conclusions of this article will be made available by the authors, without undue reservation.

## Ethics Statement

The studies involving human participants were reviewed and approved by Institutional (University Hospital Heraklion) Ethics Committee approval (1007, 33/2.12.2020). The research regarding systemic documentation of inherited arrhythmogenic cardiovascular diseases among Cretan children is the topic of PhD study (School of Medicine, University of Crete, PhD candidate Dr. Alena Bagkaki). Written informed consent to participate in this study was provided by the participants' legal guardian/next of kin. Written informed consent was obtained from the minor(s)' legal guardian/next of kin for the publication of any potentially identifiable images or data included in this article.

## Author Contributions

AB, AT, EH, and IG made substantial contributions to the acquisition. AB, AT, EH, MT, FP, and IG analysis, or interpretation of data and for important intellectual content. IG drafted the work and revised it critically. All authors approved the version to be published, agreed to be accountable for all aspects of the work in ensuring that questions related to the accuracy or integrity of any part of the work are appropriately investigated and resolved, and read and approved the final manuscript.

## Conflict of Interest

The authors declare that the research was conducted in the absence of any commercial or financial relationships that could be construed as a potential conflict of interest.

## Publisher's Note

All claims expressed in this article are solely those of the authors and do not necessarily represent those of their affiliated organizations, or those of the publisher, the editors and the reviewers. Any product that may be evaluated in this article, or claim that may be made by its manufacturer, is not guaranteed or endorsed by the publisher.

## Abbreviations

LQT: Long QT syndrome
LQT3: Long QT syndrome type 3
ECG: Electrocardiogram
SIDS: Sudden infant death syndrome
SCD: Sudden cardiac death
ICD: Intracardiac defibrillator
TdP: Torsades de Pointes.
