# Supplementary material for: Mexiletine Treatment for Neonatal LQT3 Syndrome: Case Report and Literature Review
Source: Front Pediatr. 2021 Aug 24;9:674041. doi: 10.3389/fped.2021.674041 (PMC8422972; doi:10.3389/fped.2021.674041)
Supplement: Supplementary file 1 [file Data_Sheet_1.docx]

**Supplementary File 1. Mexiletine analysis**

**Mexiletine extraction from serum**

Blank serum samples were used for the preparation of the spiked samples at concentration levels 0, 250, and 500 ng/ml. In 100 ul of serum, 75 ul of methanol and 75 ul of acetonitrile were added and the mixture was vortexed for 20 s. Then, centrifugation at 14000 rpm was performed for 5 min. The supernatant was removed and 20 ul was injected into the LCMS system.

## Chromatographic parameters

## A liquid chromatography-mass spectrometry system was used for the analytical determination of mexiletine. A solvent mixture of water, 5 mM ammonium acetate (A), and acetonitrile (B) was selected as the mobile phase with a flow rate of 0.6 mL min^-1^. Separation of the mexiletine was achieved on a Discovery C18 HPLC column (250 x 4.6 mm, 5 μm) at 30^ο^C. The mexiletine eluted at 12 min. A mass spectrometer, coupled with an atmospheric pressure chemical ionization (APCI) interface and a single quadrupole mass filter, was used to detect and quantify in the selected ion monitoring (SIM) mode, in the positive mode, with ions m/z 180.05, and 221.1.
