# Supplementary material for: Mexiletine Treatment for Neonatal LQT3 Syndrome: Case Report and Literature Review
Source: Front Pediatr. 2021 Aug 24;9:674041. doi: 10.3389/fped.2021.674041 (PMC8422972; doi:10.3389/fped.2021.674041)
Supplement: Supplementary file 2 [file Data_Sheet_2.docx]

**Supplementary File 2. Mexiletine treatment for neonatal LQT3 syndrome. Review of case reports.**

|  | **Presentation** | **12-lead ECG/**  **Holter** | **QTc (max)**  **ms** | **Treatment** | **DNA testing** | **Outcome**  **(symptoms)** | **First case in family?** | **Parents’ ECG/ genetic testing** | **Reference (Reference list No)** |
| --- | --- | --- | --- | --- | --- | --- | --- | --- | --- |
| Case 1 | PB | 2:1 AVB | 560 | Mex | SCN5A  (Val1764Met) | No | Yes | Unknown/negative  Brother negative | Okuwaki, 2019 (1) |
| Case 2 | FB, PB | 2:1 AVB, TdP | 690 | Pro, Mex, Ran, Dil,Phe, PM,ICD | SCN5A  (Q1475P) | TdP | Yes | Normal/unknown | Tan RB, 2017 (2) |
| Case 3 | FB,PB | T alt, TdP | 730 | Lid, Pro, Mex | SCN5A  (C5314G) | No | Yes | Unknown/unknown | Peach Ch, 2011 (3) |
| Case 4 | FB, PB | 2:1 AVB,Talt | 612 | Lid, Pro, Mex | SCN5A  (Phe 1761 Leu) | No | Yes | normal / unknown | Howley L, 2009 (4) |
| Case 5 | FB, PB | 2:1 AVB, TdP | 630 | Pro, Mex, PM | SCN5A  Novel (Thr for Met) | No | Yes | Normal/ unknown | Sarkar, 2007 (5) |
| Case 6 | FB, PB | 2:1 AVB, TdP | 825 | Lid, Pro, Mex, Mg, PM,ICD | SCN5A  (F1473C) | No | Yes | Normal/negative | Bankston JR, 207 (6) |
| Case 7 | FB, FT, PB, PT | VT, TdP, AVB | 740 | Pro, Mex, PM | SCN5A  ( P1332L) | No | Yes | Unknown/negative | Kehl HG, 2004 (7) |
| Case 8 | FB, PB, VT | 2:1 AVB, TdP | 611 | Lid, Mex, Pro | SCN5A  (V1763M) | SCA, SCD | Yes | Normal/negative  Sister negative | Chang CC, 2004 (8) |
| Case 9 | PB, VT | 2:1 AVB, TdP | 580 | Lid, Pro, Mex | Not performed | No | No | LQTS family  SCD sister and sister of mother | Yao CT, 2002 (9) |
| Case 10 | HF, SCA | TdP, T alt | 495 | Pro, Mex, LCSD | SCN5A  (R1623Q) | SCA | Yes | Normal/negative  Brother negative | Miura M, 2003 (10) |
| Case 11 | VT | TdP, T alt | 600 | Pro | SCN5A  (Ala1330Pro) | SCD | Yes | Normal/negative  Brother negative | Wedekind H, 2001 (11) |
| Case 12 | Postnatal ECG | T alt | 560 | Pro, Mex | SCN5A  (Tyr1795Cys) | No | No | LQT3 family  mother, sister of mother, mother of mother | Current case |

FB – fetal bradycardia, FT – fetal tachycardia, HF – hydrops fetalis , PB – postnatal bradycardia, PT – postnatal tachycardia, AVB – atrioventricular block, Talt – T wave alternans, TdP – Torsades de Pointes, VT – ventricular tachycardia, SCA – sudden cardiac arrest, SCD – sudden cardiac death, Pro – propranolol, Mex – mexiletine, Lid – lidocaine, Mg – magnesium, Ran – ranolazin, Dil – diltiazem, Phe - phenytoin , LCSD – left cardiac sympathetic denervation, PM – pacemaker, ICD – implantable cardioverter-defibrillat

**References**

1. Okuwaki H, Kato Y, Lin L, Nozaki Y, Takahashi-Igari M, Horigome H. (2019) Mexiletine infusion challenge test for neonatal Long QT syndrome with 2:1 Atrioventricular block. Journal of Arrhythmia 35: 685-688
2. Tan RB, Chakracarti S, Busovsky-McNeal M, Walsh A, Cecchin F. (2017) Compleity of ranolazine and phenytoin use in an infant with long QT syndrome 3. Heart Rhythm Case Reports Vol3,No1
3. Peach C, Suchowerskyj P, Gebauer RA. (2011) Succesful treatment of a newborn with genetically confirmed long QT syndrome 3 and repetitive Torsades De Pointes tachycardia. Pediatr Cardiol 32:1060-1061
4. Howley L, Di Maria M, Bailey A, Schaffer MS (2010) Neonatal long QT syndrome type 3 predicted by positive lidocaine challenge. Pace 33(3): 377-9
5. Sarkar S, Brumund M, Darling R, Snyder CS. (2007) Survival of a newborn with 2:1 Atrioventricular block, Long QT syndrome, and Torsades de Pointes. Ochsner J. 7(4): 181-184
6. Bankston JR, Yue M, Chung W, Spyres M, Pass RH et al (2007) A novel and lethal de novo LQT3 mutation in a newborn with distinct molecular pharmacology and therapeutic response. PLoS ONE 2(12): e1258
7. Kehl HG, Haverkamp W, Rellensmann G, Yelbuz M, Krasemann T, Vogt J, et al (2004) ife-threatening neonatal arrhythmia.Succesful treatment and confirmation of clinically suspected extreme long QT syndrome 3. Circulation 109:e205-e206
8. Chang CC, Acharfi S, Wu MH, Chiang FT, Wang JK, Sung TC et al (2004) A novel mutation manifests as a malignant form of Long QT syndrome with perinatal onset of tachycardia/bradycardia. Cardiovascular Research 64: 268-278
9. Yao CT, Wang JN, Tsai YC,et al (2002) Congenital long QT syndrome with functionally impaired atrioventricular conduction: successful treatment by mexiletine and propranolol. J Formos Med Assoc 101:291-3
10. Miura M, Yamagishi H, Morikawa Y, Matsuoka R. (2003) Congenital long QT syndrome and 2:1 atrioventricular block with a muttion of the SCN5A gene. Pediatr Cardiol 24:70-72
11. Wedekind H, Smits JP, Schulze-Bahr E, Arnold R, Veldkamp MW, Bajanowski T, et al (2001) De novo mutation in the SCN5A gene associated with early onset of sudden infant death. Circulation 4;104(10):1158-64
